# NightShift: NMR shift inference by general hybrid model training - a framework for NMR chemical shift prediction

**DOI:** 10.1186/1471-2105-14-98

**Published:** 2013-03-16

**Authors:** Anna Katharina Dehof, Simon Loew, Hans-Peter Lenhof, Andreas Hildebrandt

**Affiliations:** 1 Center for Bioinformatics, Saarland University, 66041 Saarbrücken, Germany; 2 Johannes-Gutenberg-University Mainz, 55128 Mainz, Germany

## Abstract

**Background:**

NMR chemical shift prediction plays an important role in various applications in computational biology. Among others, structure determination, structure optimization, and the scoring of docking results can profit from efficient and accurate chemical shift estimation from a three-dimensional model.

A variety of NMR chemical shift prediction approaches have been presented in the past, but nearly all of these rely on laborious manual data set preparation and the training itself is not automatized, making retraining the model, e.g., if new data is made available, or testing new models a time-consuming manual chore.

**Results:**

In this work, we present the framework NightShift (NMR Shift Inference by General Hybrid Model Training), which enables automated data set generation as well as model training and evaluation of protein NMR chemical shift prediction.

In addition to this main result – the NightShift framework itself – we describe the resulting, automatically generated, data set and, as a proof-of-concept, a random forest model called Spinster that was built using the pipeline.

**Conclusion:**

By demonstrating that the performance of the automatically generated predictors is at least en par with the state of the art, we conclude that automated data set and predictor generation is well-suited for the design of NMR chemical shift estimators.

The framework can be downloaded from https://bitbucket.org/akdehof/nightshift. It requires the open source Biochemical Algorithms Library (BALL), and is available under the conditions of the GNU Lesser General Public License (LGPL). We additionally offer a browser-based user interface to our NightShift instance employing the Galaxy framework via https://ballaxy.bioinf.uni-sb.de/.

## Background

Nuclear Magnetic Resonance (NMR) chemical shift prediction has developed into a valuable tool for computational structural biology and biomolecular NMR spectrometry. While the high dependency of NMR chemical shifts on structural details renders their prediction a formidable task, it simultaneously makes them a very valuable source of structural information for applications like structure determination and optimization [1-4], and the scoring of protein-protein docking results [5-7]. Hence, a variety of fields profit from efficient and accurate chemical shift estimation from a three-dimensional model of the molecule under consideration – in docking, for instance, predicted shifts for the candidate complexes are compared to the experimentally observed ones as a measure of reconstruction error.

The development of novel NMR chemical shift prediction techniques is a challenging task. Previous approaches either focus on full quantum mechanical models (e.g. [8-11]) which are computationally very expensive, or settle for so called semi-classical approximations borrowed from classical physics [5,12-14]. As a third option, prediction techniques can use statistical models based on semi-classical, structural, or sequential features of the proteins (e.g. PROSHIFT [15], ShiftX [14,16], SPARTA [17], CamShift [18], BioShift [19], SPARTA+[20], ShiftX2 [21], or shAIC [22]). For high-throughput applications, the most successful approaches today offer good prediction accuracy with relatively low computational cost by combining semi-classical and statistical approaches. These techniques are known as *hybrid *methods.

Developing a new hybrid method, or extending an existing one, is a hard and complex task for which three questions have to be addressed: which features should be included into the model, which statistical technique should be employed, and which data set can be used.

The question of data set generation in particular is a very difficult one. The required information for creating such a data set is spread over several data bases, such as the Biological Magnetic Resonance Bank (BMRB) [23] and the Protein Data Bank (PDB) [24] and is stored in different, notoriously hard-to-parse, file formats. To make matters worse, real-life data sets often contain serious syntactical, semantical, and logical errors or inconsistencies. Consequently, a number of publications [21,25-29] discuss the necessity of checking and correcting the given data, BMRB NMR data and PDB coordinates alike. Typical issues for creating NMR chemical shift prediction data sets are the completeness and quality of PDB files, the chemical re-referencing problem for NMR data, and the exclusion of homologs within the data set. For BMRB files, a number of approaches (e.g. [25-28]) have been developed to detect and correct assignment and referencing errors. Mainly due to these complications, most former approaches rely on hand-curated data sets created by the application of unstandardized sequences of restriction and correction methods.

Another challenge when training prediction models is the computation of the semi-classical terms, or the structural and sequential features to learn from. Computing these terms and molecular features correctly, reliably, and efficiently requires complex molecular data structures and algorithms. Further technical challenges are the computation and the choice of a statistical model.

In this work, we present an extensible automated framework called *NightShift* for data set generation and training of hybrid NMR chemical shift prediction methods. Most importantly, typical semi-classical terms for shift prediction are implemented and readily available. As of now, we include random coil contributions, aromatic ring current effects, electric field contributions, and hydrogen bonding effects. In addition, the feature set for the training of the statistical term encompasses sequential, structural (angles, surface, and density), force-field based, as well as experimental properties. All features are computed using our open source library BALL [30], and can be easily extended.

Due to its modular nature, the framework can employ data for both, protein structures and chemical shifts, from a variety of sources. As long as the input is available in the form of one of several recognized standard file formats (e.g., PDB for the proteins and NMRStar for the shift data), NightShift can easily train and evaluate models on it. As demonstrated in this paper, this freedom can, e.g., be helpful in addressing some of the current controversies in shift prediction. For instance, the user can freely decide on a shift reference correction method of his choice, or validate models trained on non-reference corrected data sets on rereferenced ones, or study the difference of models trained on X-Ray-derived protein structures to those based on NMR-derived ones.

To demonstrate that the data collected by the framework is indeed of use for NMR shift prediction, we train and evaluate a simple hybrid prediction model. The whole training and evaluation process is completeley automated and does not require human intervention. Based on recent research [16,21], we choose a random forest model for the statistical contribution of this proof-of-concept predictor which is known for its prediction quality and efficiency, and in our experiments has demonstrated to yield very accurate and stable results. In general, however, the pipeline is model-agnostic and can be used with any regression technique implemented in R [31].

## Methods

A framework as complex as NightShift depends on a reasonable data model that can be easily accessed during all stages, as well as be easily extended.

To this end, we store all accumulated information that is needed for the training of our chemical shift prediction model in later stages, namely the experimental shifts of the atoms, the corresponding atomic features, and the filter or quality scores, in an SQLite data base (http://www.sqlite.org).

The underlying data model consists of two tables: a PDB-BMRB-chain mapping and an atomic property table, focusing at individual chemical shifts and the computed atomic features.

### Data sources

In principle, a number of data sets for NMR protein chemical shift prediction is available, e.g., the recent ShiftX2 [21] training and test set, the RefDB [25], the PROSHIFT set [15], the TALOS+ set [32], or the general PDB to BMRB mapping of the BMRB [23] itself. Apart from the RefDB and the BMRB mapping tables, the data sets were hand crafted in a time-consuming manual process. To the best of our knowledge, all previously proposed prediction approaches created such a data set manually, using many individually chosen filtering criteria. This, however, has three major disadvantages: first, it is a very cumbersome and time-consuming process that is usually not repeated when new experimental information becomes available. Second, the manual curation of the data set may impose a certain bias into the final models. And third, cutting away much of the input space might remove valuable information. The first problem could be circumvented by using one of the available data sets (some information about these data sets can be found in Table 1). However, all of these suffer from the other two mentioned problems.

**Table 1:** Summary and comparison of data sets used by different hybrid shift prediction approaches

**Approach**	**Year**	**Size of training set**	**Size of test set**	**X-ray/NMR resolved**	**Homolog**
		**Files (shifts)**	**Files (shifts)**		**Exclusion**
Meiler [15]	2003	292 (n. a.)	30 (n. a.)	*mixed*	N
Sparta [17]	2007	200 (n. a.)	25 (n. a.)	X-ray	N
CamShift [18]	2009	n. a. (224,036)	35 (n. a.)	X-ray	N
ShiftX2 [21]	2010	197 (206,903)	61 (n. a.)	X-ray	Y
NightShift	2012	515 (326,652)	344 (217,768)	*NMR, X-ray, or mixed*	Y

The third and fourth columns show the size of the data set, measured as the number of proteins contained within. The CamShift publication reported only the overall number of shifts, which we denote in parentheses. Note that for the NightShift set, we already excluded homologous proteins. The table only includes methods for which these numbers could be reliably determined.

Thus, we decided to approach the problem differently in our pipeline: we want to make use of all available data with the ability to easily retrain the model. To obtain the largest possible data set available at any given moment, we make use of the official mapping between PDB and BMRB entries provided by the BMRB.

Former approaches to NMR chemical shift prediction typically rely on X-ray resolved structures as input while neglecting NMR resolved ones. This might seem unintuitive at first: if we have access to experimental shift information for a given protein, we typically also find a protein structure that was resolved using NMR, based on exactly those shifts under the exact same experimental conditions. This structure, though, is typically ignored in training a shift prediction method, and instead replaced with an alternative X-ray resolved one for the same – or, more typically, a highly homologous – protein. The main reason for this choice is structure quality: if both, X-ray and NMR structures exist for the same protein, the X-ray resolved ones typically feature better resolution, which is considered crucial for shift prediction.

On the other hand, the physico-chemical conditions in a protein crystal differ strongly from those encountered in the NMR experiment in which the chemical shifts were measured. In fact, there is evidence for statistically significant deviations between NMR and X-ray structures for the same protein that often exceed experimental uncertainties and RMSDs within the NMR ensemble [33]. In addition, proteins are often modified for crystallisation purposes, including amino acid mutations. In many instances, the sequence of the protein studied in the NMR experiment differs from that used during crystallisation.

Hence, the usage of X-ray resolved structures in combination with experimental chemical NMR shifts as a training set for a prediction technique is at least debatable: while X-ray typically yields higher general structure quality and resolution, the crystallized conformation may differ from the one seen in the NMR experiment by an amount exceeding the difference in resolution.

An automatic framework such as the one described in this work is obviously ideally suited to study questions such as these: the modular design allows to exchange the protein data source, such that the user can easily switch between X-ray and NMR-resolved structures, can compare the generated models, or evaluate models trained on the one set on the other. As a first step, and a proof-of-concept, we hence decided to study whether NMR-derived data sets support the training of predictors of comparable performance to those trained on X-ray derived ones.

In addition to the intrinsic consistency, the choice of NMR resolved structures relieves us from deducing missing hydrogen positions in many cases. Hydrogen placement not only affects and biases the hydrogen prediction but also the predictions for other atom types, since hydrogen atoms are present everywhere and form hydrogen bonds, an important feature in most prediction approaches. Instead of deducing them from other algorithms, NMR resolved structures directly contain this information.

An alternative source for the shift data with similar advantages to the BMRB is the RefDB, which is essentially a referencing-corrected version of the BMRB. As discussed in [25], there is sufficient reason to believe that a non-negligible percentage of chemical shifts in the BMRB have been misreferenced, which can obviously lead to spoiled shift prediction. To demonstrate the versatility of our framework, we decided to train a model on both, raw and reference corrected data. The model trained on raw data can help to assess how well automated predictor generation from non-rereferenced data can perform as a lower bound for the achievable accuracy. By comparison to the results on referenced data, our framework can then additionally be employed to study the influence of reference correction on prediction quality, or to analyze the question of potential bias in the reference corrected data. Comparative studies such as these are greatly simplified by the modular design of our pipeline, where the data download step can be easily adapted to query the data from varying sources.

### Restricting the data

To create a reasonable data set from the primary data sources, we apply two types of filters: type-1 filters restrict the initial BMRB to PDB mapping, while type-2 filters evaluate individual molecule files.

When creating a protein-only model, we want to skip all PDB entries containing additional ligands or DNA (NightShift in principle allows the creation of other kinds of prediction models, too (c.f. [34]), but this is out of scope of this work). To reliably decide whether a PDB entry contains a ligand before downloading and parsing the file itself, we query the PDB RESTful web service (http://www.rcsb.org/pdb/software/rest.do) to parse its results.

Furthermore to exclude mismatches, mutations, and improbable structural data we demand an alignment score of 100% between the residue sequence in the BMRB and the PDB file. To this end, we employ ClustalW [35].

To avoid overfitting through homolous structures in test and training set, we cull the mapping by sequence similarity with a cutoff of 10% by applying the standalone PISCES package provided by the Dunbrack group [36].

To restrict the data set to reasonable and trustworthy data, we apply type-2 filters, namely the structure’s Amber energy [37] with a default cutoff value of 1000 kJ/mol.

### Data mapping

Reading the BMRB data correctly can be challenging. For mapping atomic to experimental data we follow a two step procedure. First we employ ClustalW [35] to map the residues between PDB and BMRB, followed by BALL’s PDB-to-NMRStar name converter to identify corresponding atoms. While computing the atom mapping we encountered the following problems: first, both data bases employ different naming and index conventions and second, both use non-unique chain identifiers.

We solve the problem of non-unique chain identifiers by using the results of our alignment procedure to re-index the chains. If multiple chains can be matched with equal best alignment score, we simply choose the first match.

### Choice of statistical models

This work focuses more on the creation of a pipeline for automated predictor generation than on fine-tuning the parameters of the resulting predictors themselves. Hence, we relied on former research for the choice of a statistical model.

[16,21] studied different learning techniques for protein NMR chemical shift prediction based on sequential and structural features, and both suggested bagging learners. We thus decided to apply Random Forests [38], which combine bagging with feature selection and belong to the class of bootstrap aggregating machine learning techniques. A key feature of random forests is the randomized generation of subset training data for each tree in the forest, which protects the final model from suffering high error variance. Thus, the random forest method is a variance reduction technique and as such considered to be resistant to overfitting.

For training the prediction models, we decided to use R [31], the de-facto standard for open source statistical computing. Random forests were trained using the R package randomForest [39] with 500 trees to grow and all variables sampled as candidate at each split. m _*try*_ was determined using the recommended tuning procedures.

### Separation of models by atom type

A final decision before training the models concerns the handling of different atom types. As extreme cases, all atom types could be collected into one large model that contains the type as one additional feature, or different models could be trained for each atom type for which a significant number of experimentally determined shifts are available.

The first option is, to the best of our knowledge, never used in chemical shift prediction: the physico-chemical processes that govern the influence of features (such as torsion angles or sequence neighbourhood) on an atom’s shift value differ too widely to combine them into a single formula. The other extreme, on the other hand, might lead to the risk of overfitting the models, in particular for the hydrogens, which can be classified into many different atom types. Using Amber [37] atom types, for instance, we would end up with 32 individual models for all protein H, N and C atoms. For comparison, the ShiftX2 approach employs 6 backbone and 34 side chain models.

In our study, we decided for an intermediate approach. We thus designed the concept of atom super classes, which cluster force-field based atom types to form subsets of comparable size whenever possible. This clustering can be either defined by the user or automatically generated by the framework based on the data set at hand. In our experiments, we found a set of 10 types to be suitable, for which we then trained and evaluated separate models. The final atom super classes are given in Table 2. Please note that the sizes for each super class in the table were taken from the ‘raw’ BMRB set, but do not vary strongly on the RefDB-set.

**Table 2:** Definition and number of data points for our atom super classes using notation borrowed from the Amber force field

**Atom super class**	**Description**	**Shifts**
N	Backbone nitrogen	65,246
CA	Alpha backbone carbon	66,579
CB	Beta backbone carbon	60,353
C	Backbone carboxy carbon	48,442
H	Backbone hydrogens attached to the backbone nitrogen	68,461
HA	Side chain hydrogens on alpha positions (HA, 1HA, 2HA)	71,066
HB	Hydrogens on beta positions (HB, 1HB, 2HB)	62,106
HD	Hydrogens on delta positions (2HD, HD1, HD2, 1HD1, 1HD2, and 2HD2)	37,514
HG	Hydrogens on gamma positions (HG, 1HG1, 1HG2, 2HG, 2HG1, HG1)	43,221
HEHZ	Remaining hydrogens (HE, HE1, HE2, HE3, 2HE, 1HE, 1HE2, 2HE2, HH2, HZ, 1HZ, HZ2, HZ3)	21,532

### Performance evaluation of the models

The careful analysis and evaluation of the generated model is arguably the most crucial step in predictor generation. We hence decided to include functionality for automated conservative evaluation directly into the pipeline. To this end, we first choose an independent test set at random from the input data set, which is removed from the training set. In a data rich situation as the one described in this work (c.f. Table 2), such a complete separation of training and test set is typically prefered to alternative approaches for approximating the generalization error, such as cross validation or bootstrapping [40]. For a conservative evaluation, it is greatly advisable to perform homology restriction in the pipeline, as described earlier in this work. Comparison to state-of-the-art techniques was performed by applying the stand-alone version of ShiftX2 on our test data set (c. f. Table 3).

**Table 3:** Performance (correlation coefficients and rmse) of our models in comparison to ShiftX2 using the test set created by our pipeline

**Prediction**	**N correlation**	**CA correlation**	**CB correlation**	**C correlation**	**H correlation**
**Method**	**(rmse)**	**(rmse)**	**(rmse)**	**(rmse)**	**(rmse)**
Spinster	0.817 (2.977)	0.956 (1.425)	0.992 (1.582)	0.731 (1.524)	0.593 (0.505)
ShiftX2	0.554 (5.606)	0.953 (1.475)	0.984 (2.238)	0.711 (1.65)	0.534 (0.583)
Training / test size	39,147 / 26,099	39,947 / 26,632	36,211 / 24,142	29,065 / 19,377	41,076 / 27,385
Prediction	HA correlation	HB correlation	HD correlation	HEHZ correlation	HG correlation
method	(rmse)	(rmse)	(rmse)	(rmse)	(rmse)
Spinster	0.997 (2.889)	0.82 (0.559)	0.994 (0.324)	0.981 (0.375)	0.86 (0.365)
ShiftX2	0.517 (5.998)	0.721 (1.033)	0.012 (0.706)	0.816 (0.505)	-0.147 (0.967)
Training / test size	42,639 / 28,427	37,263 / 24,843	22,508 / 15,006	12,919 / 8,613	25,932 /17,289

The size is measured in the number of available atomic shifts.

The test errors of our models can be estimated from the root mean square error (rmse) and Pearson’s Correlation Coefficient (corr):

(1) $$\begin{array}{ll} \text{rmse} & =\sqrt{\frac{\overset{n}{\underset{i=1}{\sum }}{\left(\delta_{i}^{\text{exp}}-\delta_{i}^{\text{pred}}\right)}^{2}}{n}}\; \end{array}$$

(2) $$\begin{array}{ll} \text{corr} & =\frac{1}{n-1}\overset{n}{\underset{i=1}{\sum }}\left(\frac{\delta_{i}^{\text{exp}}-{\hat{\delta }}_{i}^{\text{exp}}}{s_{\delta^{\text{exp}}}}\right)\left(\frac{\delta_{i}^{\text{pred}}-{\hat{\delta }}_{i}^{\text{pred}}}{s_{\delta^{\text{pred}}}}\right)\; \end{array}$$

with *δ*^exp^ denoting the experimentally measured chemical shift of an atom, *δ*^pred^ the predicted chemical shift, and *n* the number of predictions made. ${\hat{\delta }}^{\text{exp}}({\hat{\delta }}^{\text{pred}})$ denotes the mean of the experimentally measured (predicted) shifts and $s_{\delta^{\text{exp}}}$$\left(s_{\delta^{\text{pred}}}\right)$ its standard deviation.

## Results

The two main results of this work are the pipeline and the generated data sets. In the following, we present these in more detail. In addition, we discuss – as a proof-of-concept – a new shift prediction model (called Spinster – Single ProteIn NMR Shift deTERmination) that was automatically generated by the pipeline.

### Pipeline

**Figure 1:**
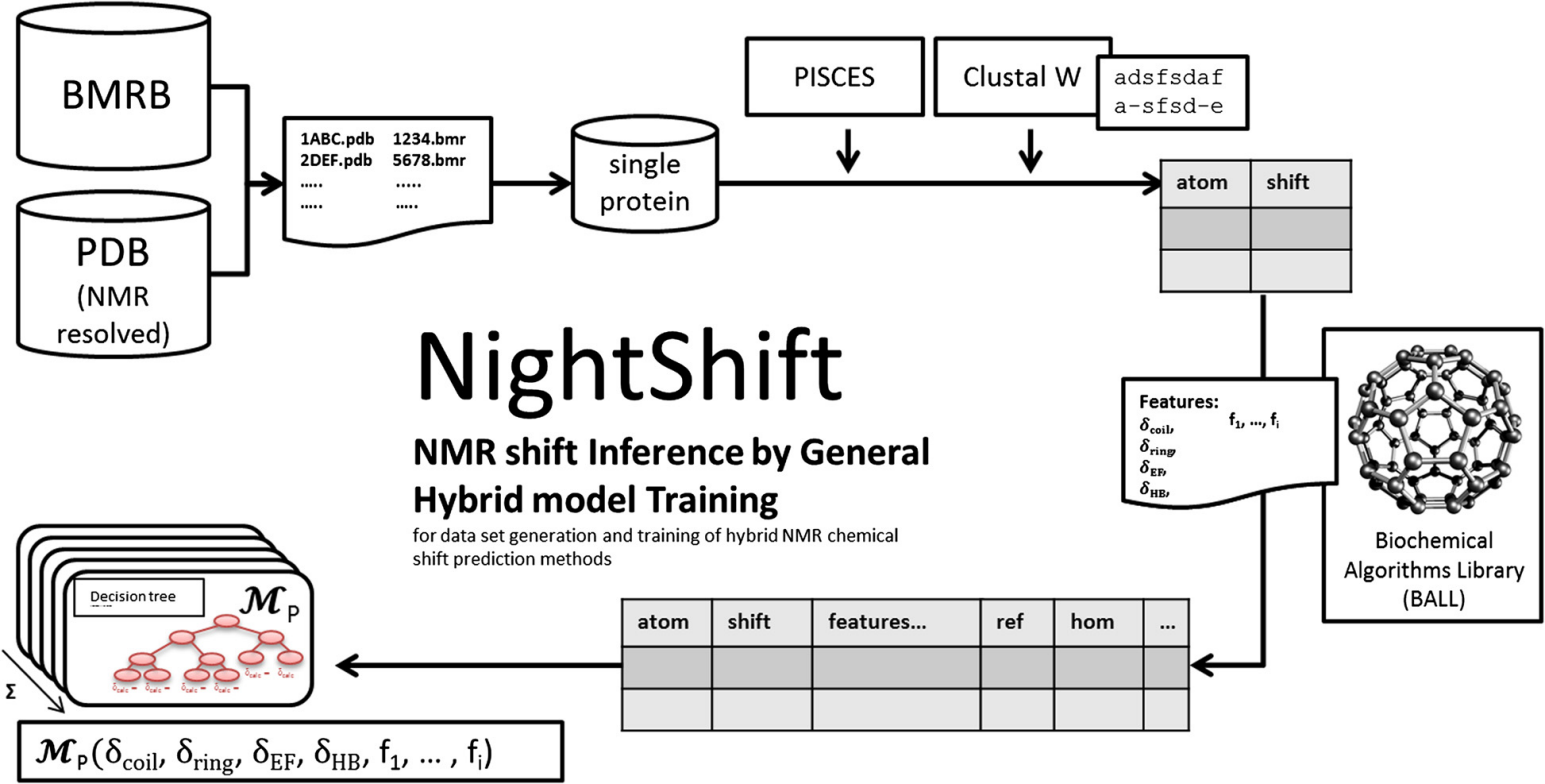
Our NightShift pipeline for data set generation and training of NMR chemical shift prediction models.

Our key goals in developing NightShift were its automizability, flexibility, robustness, and simple extensibility – goals that can easily become contradictory if they are not carefully addressed. By comparing several manual NMR chemical shift prediction approaches, we identified the following steps: (a) creation of an initial mapping between NMR and structural data, (b) filtering and restriction to a high-quality and non-homologous subset, (c) linking the NMR information to individual atoms, (d) computing the proposed features and storing them in a format that can be easily queried, and (e) training and evaluating the proposed statistical model. Figure 1 shows our proposed workflow. We now describe the pipeline in more detail.

#### Creating an initial experiment-to-structure mapping

The very first task in our pipeline is the construction or selection of a reasonable BMRB to PDB mapping. As described in section “Data sources”, the BMRB is regularly updated, is the largest of the possible sets, provides unbiased data and high consistency between structural and chemical shift information. We thus decided for the BMRB mapping as inital mapping.

To automatize data set creation, which allows retraining as soon as new data becomes available, the pipeline starts with a Python script that automatically queries the BMRB for its mapping and parses the results to yield a PDB-ID to BMRB accession number mapping. This step can be easily adapted to use different sources of chemical shift data. In our experiments, e.g., we also used reference-corrected shifts as deposited in the RefDB. Since this data base also offers its content in the form of NMRStarFiles, adapting the pipeline was trivial and mainly consisted in changing a single URL. This initial mapping then forms the input for the next step of our pipeline.

#### Filtering the experiment-to-structure mapping

The initial mapping needs to be further restricted with respect to the exclusion of homologs, the application of quality criteria, and the limitation to single protein entities measured in the experiment.

The pipeline first applies type-1 filters: the user can select to focus on pure protein instances and apply a user defined homology criterion, e.g., maximally 10%.

If a homology filter is not desired by the user, it can simply be skipped. Similarly, it is easily possible to extend the filtering procedure by user generated scripts. If filtering is required at a later point in the pipeline, special filter columns can be added to the created data base by a provided Python script.

The type-2 filters, the limitation to high-quality data, cannot be adressed at this point since they require the download and parsing of PDB and BMRB input data and are thus handled in a later step.

The input for the next step is then the resulting filtered mapping file.

#### Download of PDB data, BMRB data, and creation of the shift-to-atom mapping table

The goal of this step is to automatically download all PDB and BMRB files specified in the mapping file, to find a mapping between PDB atoms and BMRB atoms, to perform referencing error correction, if so desired, and to store the resulting atom mapping for the later feature computation. This functionality is covered in our pipeline by a Python script as well, which starts by automatically downloading all files (PDB and BMRB) from their respective web interfaces.

The pipeline then continues by identifying first corresponding residues, then atoms and shifts. In this stage, we store the residue alignment and its alignment score – which can be later used for quality filtering – in a mapping-related table.

In addition, for each PDB–BMRB chain pair we store general information that is related solely to the PDB or BMRB entity: NMR experimental information like availability of H, C or N NMR chemical shifts, the NMR spectrometer used in the experiment, the experimental conditions like solution, pH, temperature, and pressure. The NMR information is parsed via BALL’s Python interface, PDB information can be taken from the PDB’s RESTful web service.

Now, finally having access to the NMR file, further shift re-referencing through external programs could be added to the pipeline. This step can be skipped if re-referencing is not desired, or if the data has been taken from an intrinsically reference-corrected source, such as the RefDB.

#### Choice of input features

The choice of features to be provided to the statistical model is governed by a feature definition file. Besides some background descriptors needed for administrative issues (PDB id, chain id, residue id, Amber atom name, and experimental shift) we offer a large number of features as input for the prediction model. Our framework currently provides sequential (current, pre- and successor amino acid types, sequence length, alignment score), structural (torsional angles and secondary structure elements, explicit backbone distances, solvent accessible surface contributions, atomic densities, hydrogen and disulfide bonds), force field based (atom types, Amber energies) and experimental features (presence of H, C or N shifts, temperature, pH, pressure, solution, NMR spectrometer) as well as semi-classical contributions (Random coil effect, Ring current effect, Electric field effect, and Hydrogen bond effect). A detailed definition of each feature can be found in the Additional file 1. A number of these features is already known to the community, but we developed new features as well. Performances of all features are described in the Additional file 1 as well.

By commenting out lines in this feature definition file, pre-defined features can be easily excluded. Feature implementation is greatly simplified by BALL, which offers data structures and pre-implemented functionality for such tasks. In our pipeline, each feature is represented by a string that can be easily added to the list of features to compute. A C++ class connects these strings to functions that compute these features.

#### Computation of an atomic property table

For each matched atom, the experimental NMR chemical shift has to be linked to the corresponding structural atom data and to the atom’s features to allow for training a prediction model. To this end, the pipeline parses the previously downloaded BMRB and PDB files for the relevant information to compute and store all features, and to assign the experimental chemical shifts to PDB atom entities.

Based on the mapping table, the corresponding PDB and BMRB files (in PDB and NMRStar format, respectively) are read in and a name converter is used to identify corresponding atom entities. When addressing the assignment of experimental chemical shifts to PDB atoms, we face some technical problems since BMRB files are hard to parse correctly and in some cases, the files contain serious syntax errors or inconsistencies. We thus designed a fault-tolerant NMRStarFile parser, which we included into the BALL library, as well as data structures and algorithms for mapping and assignment.

The output of this step is a SQLite data base with two tables, one for the PDB chain to BMRB mapping and related information, the other for the atoms and their shifts and properties.

#### Training and evaluation

Finally, the NightShift pipeline reads the SQLite data base into an R-data frame and automatically trains a random forest model for each atom super class separately. The underlying R training script automatically and randomly splits the provided shift data set into a training and test sets according to a user defined ratio (we used a ratio of 60:40) and calls the statistical model’s training method. The training method is given the training data subset defined by predefined feature columns and the column to train against, and returns a vector of models, one for each atom super class.

Finally, our script automatically stores the created models in “R.data” format.

For evaluation purposes, the resulting prediction models are automatically applied to the test set and for each atom super class, the root mean square error (rmse) and Pearson’s Correlation Coefficient (corr) between experimentally measured and predicted values are computed.

#### Application of the model

The framework additionally offers an interface to apply the created models to proteins given in PDB format. Given the feature definition file, this script applies the preparation steps used for generating a query sqlite data base containing the specified features, and applies a provided R model. The output is a csv file.

#### Extensibility

The presented pipeline can be easily extended in several different directions: 

• *Re-training the models:* Given new data in the BMRB, only the PDB to BMRB mapping is needed as input to generate a new random forest model.

• *Using alternative data sets:* Modifying the pipeline to use different data sources is typically simple, in particular if the data comes in a form already understood by BALL. For instance, reference-corrected shift data can be used by either downloading the NMRStar files from a re-referenced data base, such as the RefDB, or by applying a re-referencing tool to the downloaded data as a step in the pipeline. Similarly, a change from NMR- to X-Ray - derived structures can be achieved by using the PDB’s query functionality to find the most similar (and highest-resolution) X-Ray derived entry for a given NMR structure.

• *Adding a feature:* For adding a feature, the user has to choose a feature name string, to add the feature name string to the feature definition file, and to add a method that computes the feature value(s). The feature value(s) will then automatically be included in a column named accordingly and is made available to the training procedures.

• *Adding a model:* For testing a new statistical model, the user only has to include the corresponding R package and to wrap the correct prediction method to meet our interface definition of training methods.

### Data set

For reasons of simplicity, this section only describes the data set generated from the ‘raw’ BMRB; the numbers for the data set based on the RefDB are very similar.

The original mapping downloaded from the BMRB contained 2,029 NMR resolved PDB–BMRB pairs. Within this set, some PDB files contained multiple chains, in total 236, yielding 2,265 PDB chain–BMRB pairs. After performing a 10% homology restriction via PISCES [36], we arrived at 890 different PDB–BMRB mappings, accounting for 898 PDB chain–BMRB pairs in our data base.

Interestingly, the setting chosen for the proof-of-concept analysis performed in this work turned out to simplify matters significantly in this step: building the data set from NMR resolved structures instead of X-ray resolved ones increased the consistency of the data set considerably. For X-ray resolved PDB files, the corresponding sequence in the BMRB file often differs considerably from the one in the PDB file, while in our data set, this was only rarely the case. In fact, after pruning the non-identical pairs, the data set still contained 859 PDB chain–BMRB pairs – the minimal protein size in the set is 40 residues, the maximum 370 residues – with a total of 544,520 shifts.

Comparing this to the number of features in our model, we currently use up to 44 (c.f. Table 4), this is a data rich scenario. Table 4 shows the number of shifts and features provided and actually used for each atom super class in our Spinster model.

**Table 4:** Number of shifts and features of the data set per atom super class, evaluated on the ‘raw’ BMRB data set

	**N**	**CA**	**CB**	**C**	**H**	**HA**	**HB**	**HD**	**HEHZ**	**HG**
Orig num shifts	65,440	66,870	60,761	48,686	68,496	71,243	62,116	37,523	21,548	43,227
Orig num features	111	111	111	111	111	111	111	111	111	111
Final num shifts train	39,147	39,947	36,211	29,065	41,076	42,639	37,263	22,508	12,919	25,932
Final num shifts test	26,099	26,632	24,142	19,377	27,385	28,427	24,843	15,006	8,613	17,289
Final num features	44	40	39	44	44	44	38	43	38	38

Maximum identity between proteins in test- and training data set was below 10%. The first two lines show the numbers for the raw data set before applying the training procedure, lines three and four show the number of shifts, and the last line shows the number of features used by the models (numbers for the RefDB are similar).

### Prediction model - Spinster

Based on the given data set and the chosen atom super classes, we trained a random forest model, which we call Spinster - Single ProteIn NMR Shift deTERmination. We evaluated Spinster on a randomly chosen test set which was excluded from the training set and compared our model with the state-of-the-art NMR shift predictor ShiftX2. Training and evaluation are performed automatically in the pipeline without manual intervention.

Table 3 shows the results for the non-reference corrected test set, Table 5 for data taken from the RefDB.

**Table 5:** Performance of models (correlation coefficients and rmse) in comparison to ShiftX2 using a RefDB-based test set created by our pipeline

**Prediction**	**N correlation**	**CA correlation**	**CB correlation**	**C correlation**	**H correlation**
**Method**	**(rmse)**	**(rmse)**	**(rmse)**	**(rmse)**	**(rmse)**
Spinster-Ref	0.847 (2.426)	0.97 (1.161)	0.996 (1.093)	0.794 (1.146)	0.563 (0.393)
ShiftX2	0.844 (2.53)	0.972 (1.122)	0.995 (1.133)	0.817 (1.114)	0.578 (0.44)
Training / test size	20,038 / 13,359	17,953 / 11,969	28,742 / 19,162	12,844 / 8,563	37,912 / 25,276
Prediction	HA correlation	HB correlation	HD correlation	HEHZ correlation	HG correlation
method	(rmse)	(rmse)	(rmse)	(rmse)	(rmse)
Spinster-Ref	0.998 (2.218)	0.963 (0.232)	0.997 (0.212)	0.993 (0.24)	0.94 (0.192)
ShiftX2	0.998 (2.314)	0.964 (0.227)	0.995 (0.227)	0.992 (0.244)	0.903 (0.199)
Training / test size	20,915 / 13,944	34,521 / 23,015	15,530 / 10,354	7,962 / 5,309	21,264 /14,177

The size is measured in the number of available atomic shifts.

As clearly demonstrated by the evaluation, the automatically generated models Spinster and Spinster-ref perform at least very comparably to the established ShiftX2 approach. In the case of non-reference corrected data, the model Spinster even performs slightly better than ShiftX2 in all cases. Please note that ShiftX2 performs significantly worse on the non-reference corrected data set than on the reference corrected one. While this is not unexpected per se, the fact that it is possible to train and evaluate a model on non-reference corrected data (where training and test set are kept well separated) with very comparable quality to those on reference corrected ones at least warrants further investigation.

In addition to the prediction task, the random forest models implicitely perform a feature selection. In the case of the BMRB-derived data set, e.g., out of the 111 provided features, the forests evaluate up to 44 as important. The individual importance measures are shown in the Additional file 1. Traditional features like amino acid type and torsional angles are within the top ten scored features as expected. Some of our new features also appear in the top ten, e.g., the ‘residue SAS’ – the surface accessible area of the atom’s residue – for atom super classes C, HB, HD and HEHZ.

### Web-interface

**Figure 2:**
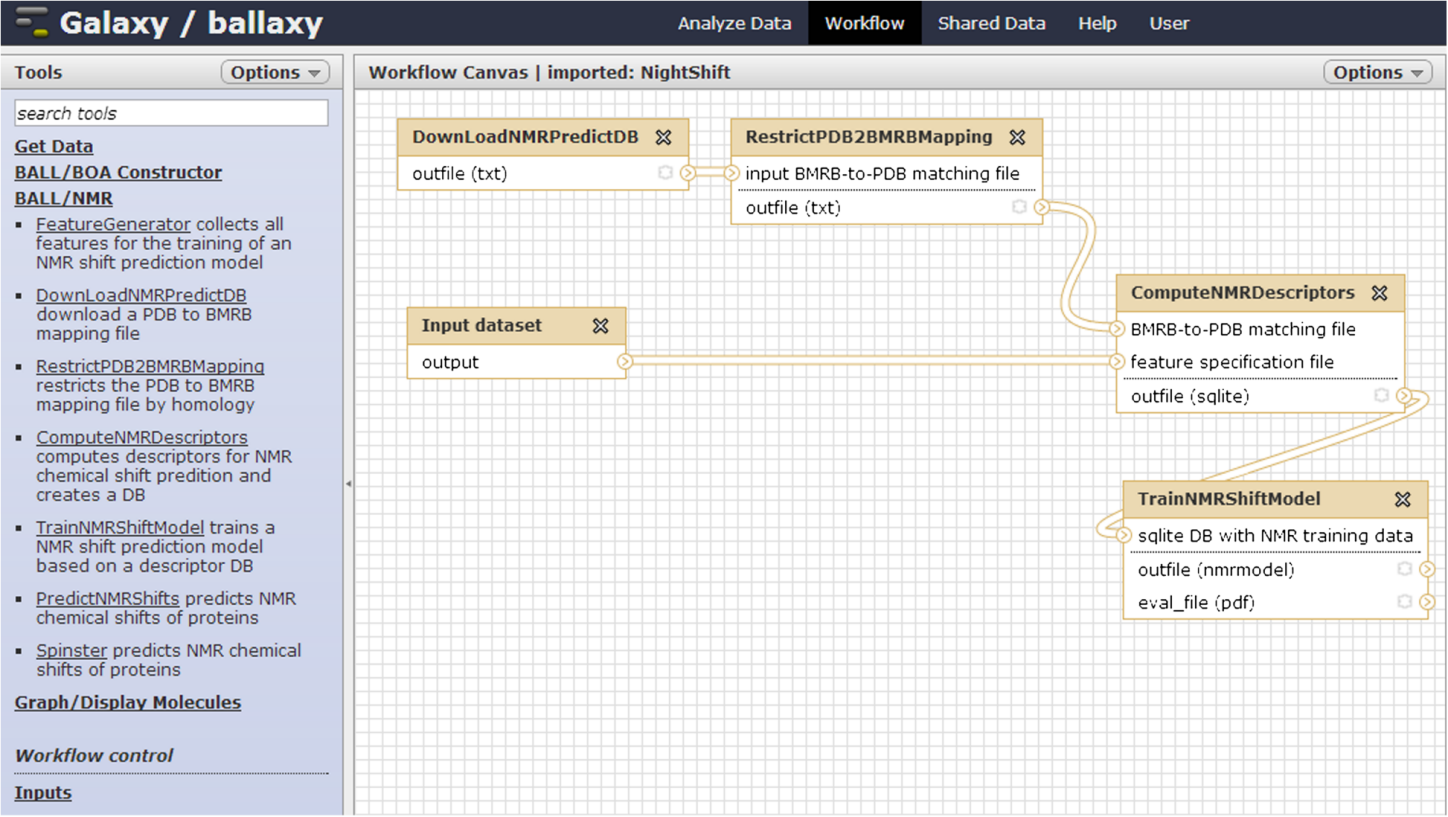
NightShift workflow via the web-interface.

To simplify the usage of our pipeline, we have created a set of tools for the ballaxy service – a web-based workflow toolkit for structural bioinformatics built on the Galaxy Workflow engine [41-43]. A manuscript on ballaxy is currently in preparation. This integration allows the user to easily generate his own prediction models for his own choice of parameters (data source, filters, features) from the browser, or to apply existing ones. Through Galaxy’s powerful workflow functionality, a user can even generate his own pipeline on demand or integrate NightShift into arbitrarily complex workflows. Figure 2 shows the web-interface.

## Discussion and conclusion

Chemical shift prediction from candidate structures plays an important role in many application scenarios in structural bioinformatics. Hence, the problem has received considerable attention in the literature. But despite tremendous advances in the field in recent years, there is still considerable room for improvement. On the one side, there is obviously the challenge to further improve the prediction quality. On the other, there is strong interest in extending the applicability to protein-DNA or protein-ligand complexes.

Unfortunately, though, the initial barrier to create a new prediction technique, potentially introducing novel features or new statistical models, is enormously high. The problems range from the seemingly trivial, but practically difficult, error-tolerant implementation of NMR file formats to the challenges in computing novel descriptors from three-dimensional structure representations.

With this work, we have contributed a framework that allows researchers not only simple access to the required data, but also a wide variety of molecular features to choose from. In addition, our reliance on open-source software packages, such as the BALL library, the R framework for statistical computing, and the SQLite data base engine, greatly magnifies the extensibility of our approach. Should a user, for instance, want to extend NMR chemical shift prediction to protein-DNA complexes, he can simply make use of the large number of molecular features available in BALL, or program his own extensions, which he can then easily feed into our pipeline.

Using our pipeline, we automatically generated exemplary data sets for training and evaluating shift prediction models based on the ‘raw’ BMRB as well as on the reference-corrected RefDB. These sets have unprecedented size and can easily grow if new data becomes available. In contrast to alternative current data sets, they have been constructed exclusively from NMR resolved structures, leading to great consistency between molecular structures and NMR shift information. Due to the unclear relative influences of structure quality and resolution on the one side, and consistency between structure and shifts on the other, the framework also allows to generate data sets based exclusively on X-ray structures, or to freely mix the two kinds. Evaluating the differences in shift prediction due to this choice will be the topic of future work, which will be greatly simplified by the NightShift-framework.

In addition to preparing the data sets, we exemplarily trained prediction models on them, again in a completely automatic fashion without any fine-tuning to further to optimize performance. Even without such optimizations, the performance of the model is surprisingly good. In fact, we find that it can easily compete with state-of-the art techniques, as tested by comparison to the well-established ShiftX2-method. First results indicate that our method seems to be particularly robust against lowly resolved structures and against the presence of wrongly referenced shifts, but further work is required before this question can be conclusively answered. Such robustness would be highly desirable for application scenarios such as molecular docking, where the input structures are merely candidates, instead of highly resolved, precise configurations.

In summary, we have demonstrated that the creation of chemical shift prediction models can be greatly simplified, and to a large extent automatized, without spoiling prediction quality. The models we presented will further improve over time with each new structure-shift pair deposited to the BMRB and RefDB and each new feature developed. Our own future work will focus on adding feature sets that are suitable for protein-ligand and protein-DNA complexes to extend the applicability of NMR chemical shift prediction to a whole new set of problems.

## Competing interests

The authors declare that they have no competing interests.

## Authors’ contributions

AH and HPL supervised the study. AKD, AH, and SL implemented the pipeline. All authors read and approved the final manuscript.

## Supplementary Material

Additional file 1 Supplementary material. Click here for file
