# Supplementary material for: NightShift: NMR shift inference by general hybrid model training - a framework for NMR chemical shift prediction
Source: BMC Bioinformatics. 2013 Mar 16;14:98. doi: 10.1186/1471-2105-14-98 (PMC3682865; doi:10.1186/1471-2105-14-98)
Supplement: Additional file 1 — Supplementary material. [file 1471-2105-14-98-S1.pdf]

1 SUPPLEMENTARY MATERIAL

name	definition
atom_name	PDB atom type
element	atomic element
aa	amino acid type
aa_prev	amino acid type of the previous residue (according to PDB indexing)
aa_next	amino acid type of the next residue (according to PDB indexing)
ϕ	backbone torsional angle involving the backbone atoms C'-N-C^{α} -C'
ψ	backbone torsional angle involving the backbone atoms N-C^{α}-C'-N
χ	side chain dihedral angle involving N-C^{α}-C^{β}-C^{γ}
$\chi 2$	side chain dihedral angle involving C^{α}-C^{β}-C^{γ}-C^{δ}
ϕ_next	ϕ angle of the next residue
ψ_next	ψ angle of the next residue
χ_next	χ angle of the next residue
$\chi 2$_next	$\chi 2$ angle of the next residue
ϕ_prev	ϕ angle of the previous residue
ψ_prev	ψ angle of the previous residue
χ_prev	χ angle of the previous residue
$\chi 2$_prev	$\chi 2$ angle of the previous residue
secondary_structure	secondary structure of the atom's residue
disulfide	flag indicating the presence of disulfide bridge
protein_size	the protein's size in number of amino acids
atom_sas	the atom's contribution to the numerical solvent accessible surface of its molecule (r=1.5Å)
atom_sas2	the atom's contribution to the numerical solvent accessible surface of its molecule (r=0.5Å)
residue_sas	the residue's contribution to the protein's numerical solvent accessible surface (r=1.5Å)
residue_sas2	the residue's contribution to the protein's numerical solvent accessible surface (r=0.5Å)
atom_density	number of atoms within a radius of 5Å
atom_w_density	atom_density weighted with the reciprocal distance to the atoms within a 5Å radius
atom_pack	van-der-Waals volume of atoms within a 5Å radius while excluding atoms of the atom's own residue using a simple spherical capping approximation for atoms crossing the 5Å boundary
atom_w_pack	atom_pack with additional weights for the volumes with the distance to the atom under consideration
dist_com	distance of the atom to the center of mass of its protein
Amber	total Amber energy
AmberVDW	van-der-Waals energy contribution
AmberES	electrostatic energy contribution
AmberTorsion	torsional Amber energy contribution
AmberStretch	Amber bond stretch energy contribution
charge	atom's charge as defined in the PARSE parameter set (Sitkoff *et al.*, 1994)
hbond	indicator for the involvement of the current atom in a hydrogen bond as acceptor or hydrogen
hbond_donor	indicator for the atom as hydrogen bond donor (carbon, oxygen, and nitrogen)
hbond_HA	indicator for the residue's hydrogen bond via H^{α}
hbond_HN	indicator for the residue's hbond via backbone nitrogen
hbond_OH_len	length of the residue's hbond via backbone oxygen
random_coil	random coil parameter set as used in Wishart *et al.* (1995)
ring_current	ring current shift contribution as formulated in the Haigh-Mallion model (Haigh and Mallion, 1972, 1979)
electric_field	shift contribution due to electric field according to Buckingham (1960) model with parameter set from Neal *et al.* (2003)
hbond_effect	shift contributions due to hydrogen bonds according to Neal *et al.* (2003)
dist_O	distance to the next oxygen
dist_N	distance to the next nitrogen
dist_CB	distance to the next C^{β}
dist_CA	distance to the next C^{α}

Table 1. Names and descriptions of features used in the NightShift framework.

feature	N	CA	CB	C	H	HA	HB	HD	HEHZ	HG
aa	**110.82**	**74.21**	12.15	**66.07**	**53.68**	**100.68**	**7.71**	**35.47**	**62.12**	4.42
aa_next	39.74	**53.35**	8.73	22.37	37.60	10.56	2.61	3.90	8.23	6.03
aa_prev	**150.84**	11.08	2.81	15.58	**46.38**	**58.26**	2.51	9.62	10.49	0.00
**Amber**	11.45	15.17	4.89	5.32	13.27	9.40	**6.77**	**15.19**	4.02	1.05
**AmberES**	10.06	10.43	5.72	2.29	12.37	5.33	0.17	4.38	5.79	1.17
**AmberStretch**	8.19	0.00	0.00	4.72	0.00	28.11	0.00	1.05	0.00	0.00
**AmberTorsion**	**49.31**	20.22	**18.98**	**28.19**	3.60	**48.96**	2.60	0.00	0.00	0.00
**AmberVDW**	33.47	**27.14**	9.78	**25.26**	**40.22**	13.62	1.63	10.76	**18.26**	8.19
**atom_density**	40.59	17.98	12.23	21.16	27.85	33.54	1.45	2.14	10.96	8.25
**atom_sas**	15.25	17.26	2.47	18.80	26.25	15.98	**6.95**	1.64	7.90	**12.93**
**atom_sas2**	36.01	25.14	7.09	**24.21**	**45.52**	16.40	1.77	2.33	9.10	**11.19**
atom_name	0.00	0.00	0.00	0.00	0.00	**123.99**	**5.40**	**20.52**	**29.70**	2.09
**atom_pack**	29.84	9.63	13.23	**24.12**	11.68	6.74	**5.72**	2.03	14.69	8.18
**atom_w_density**	26.29	14.79	11.76	14.95	15.62	18.77	1.83	**15.43**	**15.88**	**18.68**
**atom_w_pack**	22.50	12.58	8.77	23.11	14.31	19.91	2.16	2.21	**15.24**	7.04
**charge**	8.67	17.44	3.90	10.88	5.19	11.88	3.31	**23.52**	**25.15**	**164.33**
χ	41.59	**27.92**	3.25	14.89	18.25	**34.13**	1.14	8.30	5.29	2.97
$\chi2$	22.89	25.79	10.93	13.82	16.23	13.95	**5.18**	**24.41**	10.73	6.02
$\chi2$_next	14.07	13.05	9.16	6.67	10.61	4.71	0.00	0.95	5.90	**14.64**
$\chi2$_prev	30.72	0.00	0.00	5.52	10.96	14.94	0.00	8.34	0.00	0.00
χ_next	17.07	12.46	6.92	5.95	12.85	11.98	0.97	1.93	4.11	3.40
χ_prev	43.30	0.00	0.00	3.86	22.26	**40.81**	0.00	**18.64**	0.00	0.00
**dist_com**	38.89	**27.82**	**18.03**	4.74	**53.67**	10.20	1.05	8.53	8.73	**8.78**
dist_CA	9.48	14.27	11.07	7.42	8.06	13.85	**5.36**	5.67	**18.63**	**19.24**
dist_CB	**44.40**	25.21	**16.44**	11.97	26.39	**37.84**	**6.45**	2.56	15.16	**38.95**
dist_N	22.13	17.02	**13.47**	23.39	29.16	21.17	1.58	11.94	**18.08**	6.47
dist_O	7.26	6.49	**23.59**	**27.36**	34.12	22.40	**6.62**	**12.02**	5.45	**12.67**
disulfide	0.00	**46.32**	**154.34**	0.00	0.00	0.00	5.05	0.00	0.00	0.00
electric_field	0.00	17.20	0.00	0.00	17.37	0.00	0.00	0.00	0.00	0.00
element	0.00	0.00	0.00	0.00	0.00	8.38	0.00	0.00	0.00	0.00
hbond_HA	4.46	3.17	4.33	3.59	7.37	0.00	2.30	5.31	0.00	1.68
**hbond**	0.00	0.00	0.00	0.00	14.37	0.00	0.00	0.00	0.00	0.00
**hbond_donor**	13.66	3.88	0.00	0.00	0.00	16.75	0.00	0.00	0.00	0.00
hbond_effect	0.00	0.00	0.00	0.00	23.91	0.00	0.00	0.00	0.00	0.00
hbond_HN	14.76	22.52	**17.88**	13.01	14.76	14.35	0.13	1.19	6.54	7.62
hbond_OH_len	0.00	0.00	0.00	14.63	0.00	0.00	0.00	2.10	0.00	0.00
ϕ	**80.23**	0.00	0.00	23.14	**51.62**	30.71	0.00	3.28	0.00	0.00
ϕ_next	**49.49**	20.02	8.02	23.45	25.94	15.97	2.51	5.20	6.19	4.82
ϕ_prev	**51.14**	0.00	0.00	0.00	37.84	12.50	0.00	0.00	0.00	0.00
**protein_size**	9.88	16.10	3.70	12.23	9.26	3.61	0.69	1.96	1.92	1.50
ψ	**64.53**	**118.21**	**30.08**	**34.65**	**55.26**	**38.06**	0.00	5.89	4.90	8.75
ψ_next	**59.97**	**56.26**	**15.77**	**26.93**	39.51	**42.16**	1.04	0.00	8.46	3.97
ψ_prev	**212.21**	0.00	0.00	23.15	**138.20**	**151.83**	0.00	0.00	0.00	0.00
**residue_sas**	37.95	14.04	12.67	**48.43**	39.12	21.92	**8.23**	7.42	**29.60**	8.75
**residue_sas2**	25.30	**27.92**	8.84	20.47	26.09	32.06	4.89	**27.65**	**18.41**	7.46
ring_current	17.70	5.07	10.74	13.75	**52.83**	14.71	0.00	0.00	0.00	0.00
secondary_structure	33.03	**81.27**	**43.71**	**24.71**	**51.75**	18.45	2.68	**15.05**	4.72	**8.77**

Table 2. Importance of features in the random forest model Spinster per atom super class. The values are given in the usual '% increase in MSE' scale. The new features are printed in bold, the top ten scored features are printed in bold as well.

SS type	dataset	N	CA	CB	C	H	HA	HB	HD	HEHZ	HG
coil	total	46.89	47.69	47.76	52.88	46.19	50.41	47.09	44.44	41.39	44.17
coil	train	47.91	47.37	48.27	52.50	45.98	50.08	46.71	44.04	41.21	43.40
coil	test	45.38	48.21	47.05	53.40	46.52	50.93	47.67	45.07	41.68	45.30
helix	total	33.54	26.04	36.10	30.34	36.57	31.89	34.58	35.92	36.92	34.47
helix	train	31.87	26.52	35.58	31.30	36.69	32.52	35.41	36.21	36.77	34.80
helix	test	36.01	25.25	36.84	29.01	36.39	30.92	33.35	35.48	37.14	34.00
sheet	total	19.57	26.27	16.13	16.79	17.24	17.70	18.32	19.64	21.69	21.36
sheet	train	20.22	26.11	16.15	16.21	17.33	17.40	17.89	19.76	22.03	21.80
sheet	test	18.61	26.54	16.11	17.58	17.09	18.16	18.97	19.46	21.18	20.70

Table 3. Percentages of different secondary structure elements in the test- and training data sets.

REFERENCES

Buckingham, A. D. (1960). Chemical shifts in the nuclear magnetic resonance spectra of molecules containing polar groups. *Can. J. Chem.*, **38**, 300 – 307.

Haigh, C. and Mallion, R. (1972). New tables of ring current shielding in proton magnetic resonance. *Org. Magn. Reson.*, **4**(2), 203–228.

Haigh, C. and Mallion, R. (1979). Ring current theories in nuclear magnetic resonance. *Progress in Nuclear Magnetic Resonance Spectroscopy*, **13**(4), 303–344.

Neal, S., Nip, A. M., Zhang, H., and Wishart, D. S. (2003). Rapid and accurate calculation of protein 1H, 13C and 15N chemical shifts. *Journal of Biomolecular NMR*, **26**(3), 215–240.

Sitkoff, D., Sharp, K., and Honig, B. (1994). Accurate calculation of hydration free energies using macroscopic solvent models. *Journal of Physical Chemistry*, **98**(7), 1978–1988.

Wishart, D., Bigam, C., Holm, A., Hodges, R., and Sykes, B. (1995). ^{1}H, ^{13}C and ^{15}N random coil NMR chemical shifts of the common amino acids. I. Investigations of nearest-neighbor effects. *Journal of Biomolecular NMR*, **5**(3), 332.
